# Supplementary material for: MaxQuant.Live Enables Global Targeting of More Than 25,000 Peptides
Source: Mol Cell Proteomics. 2019 Feb 12;18(5):982–94. doi: 10.1074/mcp.TIR118.001131 (PMC6495250; doi:10.1074/mcp.TIR118.001131)
Supplement: Suppl Figures [file 141202_1_supp_280577_pmnnl6.pdf]

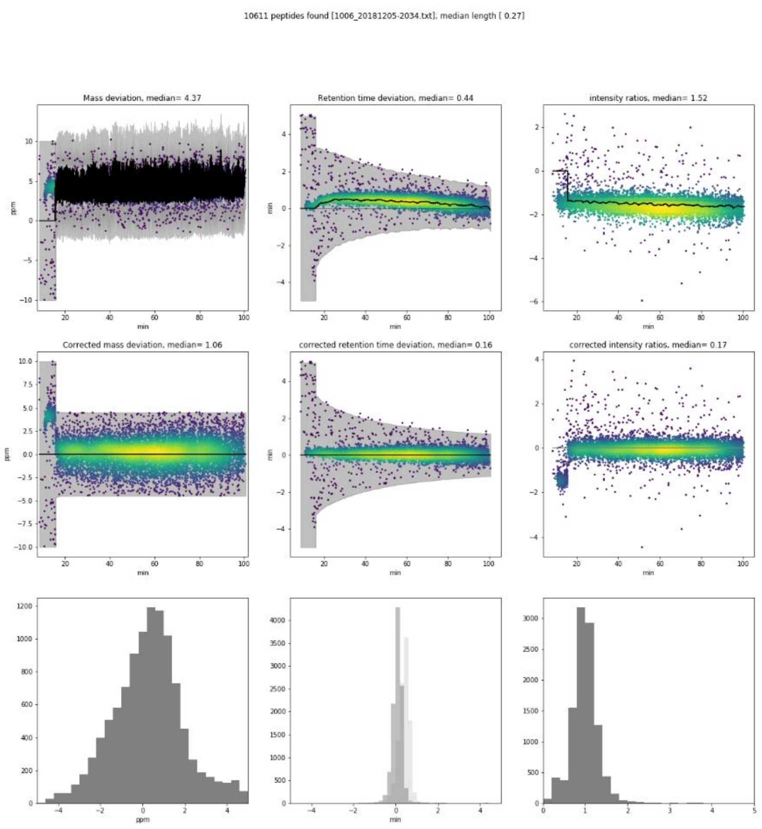

Supplemental Figure 1. Uncorrected (upper row) and corrected (middle row) deviations of recognized peptides during the targeted acquisition of breakdown curves. The columns represent the observed global shifts of the elution time (middle), mass calibration (left) and peptide intensity (right). The histograms in the lower row show the deviations from the corrected expectation values over the whole run. For the retention time deviations the histogram for the uncorrected deviations is also included (light gray).

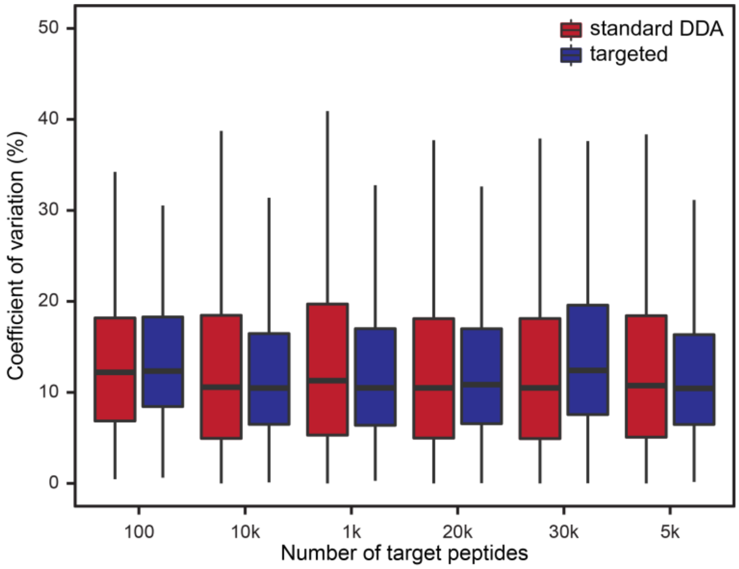

Supplemental Figure 2. Coefficients of variation in the triplicate tryptic HeLa lysate analyses in Fig. 4 for DDA and different target numbers as directed by MaxQuant.Live.
